# Petersen’s hernia after living donor liver transplantation

**DOI:** 10.1186/s40792-017-0364-5

**Published:** 2017-08-23

**Authors:** Sodai Sakamoto, Ryoichi Goto, Norio Kawamura, Yasuyuki Koshizuka, Masaaki Watanabe, Minoru Ota, Tomomi Suzuki, Daisuke Abo, Kenichiro Yamashita, Toshiya Kamiyama, Akinobu Taketomi, Tsuyoshi Shimamura

**Affiliations:** 1 Department of Gastroenterological Surgery I, Hokkaido University, Sapporo, Japan; 2 Division of Organ Transplantation, Hokkaido University Hospital, Sapporo, Japan; 3 Department of Radiation Medicine, Hokkaido University, Sapporo, Japan; 4 Department of Transplant Surgery, Hokkaido University, Sapporo, Japan

**Keywords:** Living donor liver transplantation, Petersen’s hernia, Hepaticojejunostomy, Biliary reconstruction

## Abstract

**Background:**

Hepaticojejunostomy may be used for biliary reconstruction in certain cases of liver transplantation. In this occasion, Roux-en-Y biliary reconstruction is predominantly performed. Petersen’s hernia is an internal hernia that can occur after Roux-en-Y reconstruction, and it may lead to extensive ischemic changes affecting incarcerated portions of the small bowel or Roux limb resulting in severe complications with a poor prognosis.

**Case presentation:**

The present case was a 44-year-old male who underwent living donor liver transplantation (LDLT) for familial amyloid polyneuropathy and in whom biliary reconstruction was performed with Roux-en-Y hepaticojejunostomy. Two years after liver transplantation, symptomatic bowel strangulation was diagnosed by CT examination and emergent surgery was performed accordingly. On exploration, an ischemic limb associated with Petersen’s hernia was observed. Although repositioning of the incarcerated bowel loop gradually improved the color of the limb, the limb failed to completely recover to a normal color. To allow accurate evaluation for the viability of the limb, we decided to perform a second-look operation after 48 h. On re-exploration, the surface of the limb remained a dark color; however, intraoperative endoscopic findings revealed only partial necrosis of the mucosa. Next, we resected the portion of ischemic damaged limb only following side-to-side jejunojejunostomy. Consequently, redoing of biliary reconstruction could be avoided and the original hepaticojejunostomy site was preserved. Although the stricture of the remnant Roux limb occurred 1 month thereafter, it was successfully managed by balloon dilation via percutaneous transhepatic biliary drainage route.

**Conclusions:**

The occurrence of Petersen’s hernia should always be considered in cases of liver transplantation with Roux-en-Y biliary reconstruction. On the basis of an accurate assessment of the extent of jejunal limb injury, reanastomosis of the hepaticojejunostomy, a potentially high-risk surgical procedure, can be avoided in emergent situations.

## Background

Petersen’s hernia is a complication of Roux-en-Y reconstruction and involves incarceration of a small bowel loop beneath the mesenterium of the Roux limb through Petersen’s peritoneal defect [1]. Petersen’s hernia may cause necrosis of incarcerated small bowel and ischemic injury of the Roux limb and is associated with a poor prognosis [2]. Hepaticojejunostomy with a Roux-en-Y limb is performed as part of biliary reconstruction in certain cases of liver transplantation (LT). Once this type of hernia develops in patients who underwent transplantation, it may lead to ischemic damage to the anastomosis of the hepaticojejunostomy, leading to severe complications and graft failure. To avoid the reanastomosis of the hepaticojejunostomy, which is a high-risk procedure in emergent situations, the extent of injury to the Roux limb should be accurately assessed.

## Case presentation

**Fig. 1:**
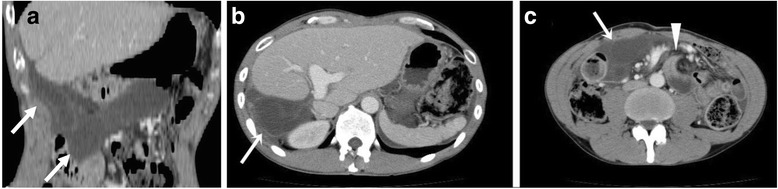
Enhanced abdominal CT scan on an arrival **a** Coronal plane. **b**, **c** Transverse plane. **a**–**c** CT demonstrated dilated, fluid-filled small bowel loops with poor enhancement of the bowel wall. Arrows indicate a markedly dilated Roux limb. c. The triangle arrow indicates a “whirl” appearance

**Fig. 2:**
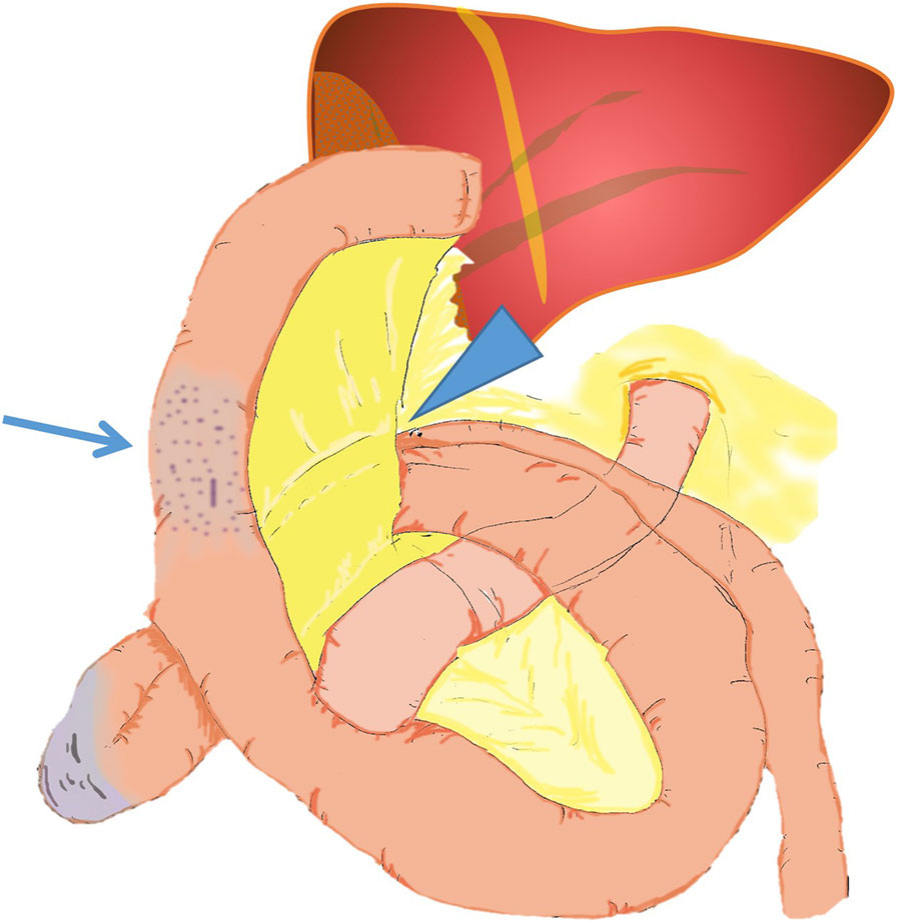
Operative findings. We identified an internal hernia through Petersen’s defect between the limb and mesocolon (*triangle arrow*). The incarcerated small bowel loop resulted in ischemia of the Roux limb (*arrow*)

**Fig. 3:**
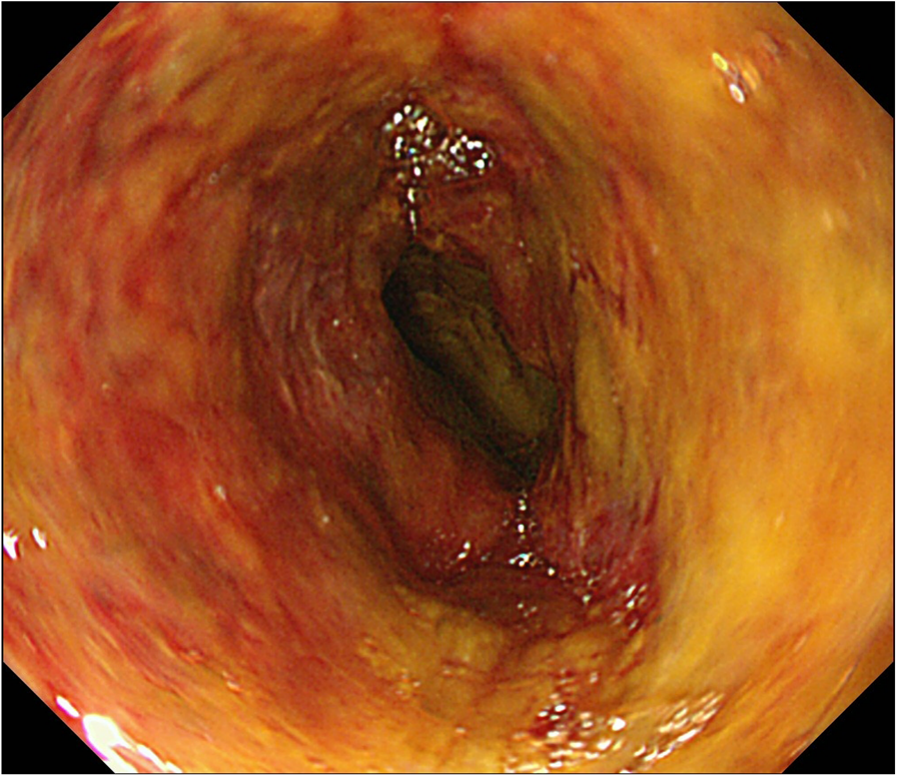
Intraoperative endoscopy view during re-exploration. Intraoperative endoscopic findings revealed only partial ischemic injury to the mucosa only

**Fig. 4:**
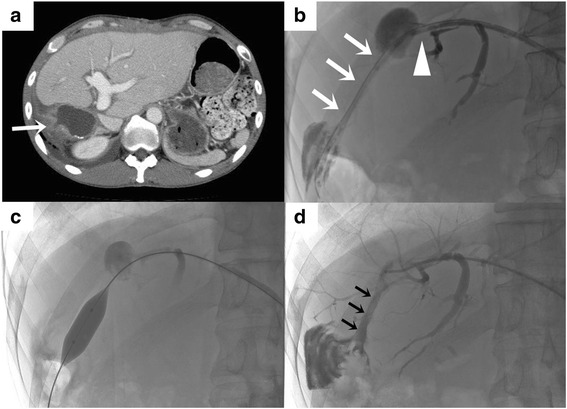
Stenosis of Roux limb at a month after discharge and the treatments. **a** CT showed dilatation of both the limb and intrahepatic bile ducts. *Arrow* indicates stenosis of the limb. **b** Tube cholangiogram through a PTBD tube of 12 Fr inserted across the stenotic lesion of Roux limb. Arrows indicate stenosis of the limb. The triangle arrow indicates the intact hepaticojejunostomy anastomosis. **c** Balloon dilation of the stenotic portion of the limb (12 mm in diameter). **d** The PTBD tube was removed 2 years after the emergency event. Arrows indicate the expanded limb

The 44-year-old man in this study was presented with sudden-onset, persistent epigastralgia and had undergone living donor living transplantation (LDLT) for familial amyloid polyneuropathy at 42 years of age, with the left hepatic lobe graft donated by his wife. During LT, biliary reconstruction was performed by hepaticojejunostomy with a Roux limb via the antecolic route as the common bile duct was removed for the sake of the following domino LT. The peritoneal defect related to Roux-en-Y anastomosis was primarily closed with several 4–0 silk interrupted sutures. Although he had experienced repeated episodes of small bowel obstruction, which had all recovered fully following conservative management, at 5, 9, and 14 months post-transplantation, continuous epigastralgia and repeated vomiting for 7 h during the present admission prompted clinical suspicion of bowel strangulation. Abdominal guarding and rigidity in the epigastric region were noted on examination. The body temperature was 37.1 °C. The blood pressure and heart rate were 132/86 mmHg and 105 bpm, respectively. The white blood cell count was elevated at 11,500/μl comprised of 89% neutrophils. However, no significant abnormalities in liver functional tests were observed. We suspected an abdominal emergency and performed an abdominal CT scan that demonstrated dilated, fluid-filled small bowel loops with poor enhancement of the intestinal wall (Fig. 1a). In particular, the Roux limb was markedly dilated (Fig. 1b). Further, a “whirl” appearance and ascites were depicted indicating small bowel volvulus (Fig. 1c). We immediately performed emergent surgery at 6 h after hospital arrival. On laparotomy, no intraperitoneal adhesions were observed. We identified an internal hernia through Petersen’s defect (Fig. 2), with a small bowel loop incarcerated beneath the mesenterium of Roux limb through Petersen’s defect causing ischemia of the limb. Following gentle manual repositioning of the incarcerated jejunum, the ischemic color of both the small bowel loop and the Roux limb gradually ameliorated. Although the former recovered completely in color, the appearance of the latter failed to regain normal color. As a treatment plan, we elected to perform a second-look operation after 48 h for precisely evaluating the condition of the limb. On re-exploration, the Roux limb remained partially dark in color indicating the possibility of remaining ischemic damage. Intraoperative endoscopic examination was performed to determine the most appropriate definitive treatment, revealing partial ischemic injury affecting the mucosa only (Fig. 3) and that the hepaticojejunostomy was intact. On the basis of endoscopic findings, we decided to perform a resection of the portion of limb affected by mucosal necrosis, followed by a jejunojejunostomy in a side-to-side fashion. Consequently a reanastomosis of the hepaticojejunostomy using a newly made limb could be avoided, a procedure that may be complicated by technical difficulty and considered a high-risk procedure in emergent settings. The postoperative course was uneventful and the patient was discharged from hospital on postoperative day 43. Unfortunately, CT imaging at 1 month after discharge showed dilatation of both the limb and intrahepatic bile ducts (Fig. 4a). We diagnosed stenosis of the limb and performed percutaneous transhepatic biliary drainage (PTBD) from a dilated terminal branch of B2 using a 7.2 Fr straight tube. Cholangiography via PTBD demonstrated segmental stenosis of the limb likely due to ischemic changes in the serosa; however, the hepaticojejunostomy appeared intact. PTBD tube was changed to 12 Fr internal-external drainage tube and placed across the jejunal stenosis (Fig. 4b), followed by balloon dilation with 12 mm diameter (Fig. 4c). Although a balloon dilation procedure was required five times, the stenotic site was successfully dilated and the PTBD tube was eventually removed 2 years after the emergency event (Fig. 4d). Recurrence of Roux limb stenosis or intrahepatic bile duct dilatation has not been observed thereafter.

### Discussion

Despite recent developments in surgical techniques, biliary reconstruction remains the “Achilles’ heel” of LT. Accordingly, Roux-en-Y or duct-to-duct biliary reconstruction is selected on an individual patient basis. Duct-to-duct biliary reconstruction is preferred even in LDLT due to easy access with endoscopy for treatment of post-transplant biliary strictures and the low incidence of refractory cholangitis [3–6]. Meanwhile, hepaticojejunostomy using a Roux limb remains an alternative option when the recipient bile duct is unusable or the liver graft has multiple hepatic ducts. Roux-en-Y hepaticojejunostomy reportedly has a lower incidence of biliary anastomotic structuring compared to duct-to-duct reconstruction in LDLT [7, 8]. In the present case report, hepaticojejunostomy was considered the only option as the common bile duct was donated with a recipient liver for a subsequent domino transplantation.

Pertersen’s hernia after Roux-en-Y reconstruction is a known complication of gastrojejunostomy for gastric cancer and bypass surgery for obesity and is observed in 0.2–6.2% of patients [9, 10]. In particular, Pertersen’s hernia reportedly occurs more frequently following laparoscopic Roux-en-Y gastric bypass surgery than open procedures [10]. Pertersen’s hernia is caused by a lack of routine closure of Petersen’s peritoneal defect and fewer abdominal adhesions after laparoscopic procedures [11–13]. Indeed, internal hernia has been rarely reported following major open surgeries such as liver-pancreatic surgery [14]. Fewer surgical adhesions after LT have also been observed, likely due to suppression of surgical adhesions by immunosuppressants [10, 11]. Blachar and Federle reported that transmesenteric hernias occurred in 20 of 43 LT cases [15]. Additionally, 48 cases of bowel obstruction in 4001 LT patients were caused by surgical adhesions in 19 cases (39.6%) and internal hernia around Roux-en-Y in 18 cases (37.5%) [16], indicating internal hernias including Petersen’s type are a major cause of bowel obstruction after LT, possibly due to fewer intra-abdominal adhesions. Indeed, no intra-abdominal adhesions were observed in the present case.

We reviewed the medical literature for cases of internal hernia following hepaticojejunostomy in LT. Table 1 summarizes the clinical features of 29 reported cases [16–21]. All cases underwent biliary reconstruction with Roux-en-Y. It is notable that internal hernias predominantly occurred around the Roux-en-Y limb. Further, 21 of 29 cases were diagnosed by CT imaging, indicating that CT assessment is a useful diagnostic tool. Twenty-seven out of 29 cases were recovered by surgery, and a further 21 cases were successfully treated with bowel repositioning followed by closure of peritoneal defects. Meanwhile, among the 6 cases requiring bowel resection, 2 patients underwent extensive resection of the small bowel resulting in a poor prognosis [18].

**Table 1:** 29 cases of internal hernia after hepaticojejunostomy in LT

Case	Author	*n*	Age (mean)	Graft	After transplantation (mean)	Hernia orifice	Treatment	Prognosis
1–18	Blacher [16]	18	12–62 (48)	Unknown	2 weeks to 10 years (14 months)	Transmesenterics or retroanastomotic	HR or BR or Graft revision	A case died despite two surgical interventions
19	Newton [17]	1	29	Unknown	Unknown	Around Roux-en-Y	HR	Alive
20–23	Khanna [18]	4	12–38 (19)	Unknown	13 days to 10 years (40 months)	Around Roux-en-Y or mesenteric window	HR or BR or small bowel transplantation	A case died after BR
24–27	Lui [19]	4	41–56 (48)	RL	19 to 23 months (20 months)	Around Roux-en-Y or mesenteric window	HR	Alive
28	Eberhardt [20]	1	12	LL	11 years	Around Roux-en-Y	HR	Alive
29	Hayashi [21]	1	42	LL	6 years	Around Roux-en-Y	HR	Alive

*RL* right lobe, *LL* left lobe, *HR* hernia repair, *BR* bowel resection

In the present case, severe complications were avoided by early diagnosis with CT and prompt exploration followed by a second-look operation in which the extent of the Roux limb injury was accurately evaluated using intraoperative endoscopic examination. Consequently, we were able to save the patient without redoing hepaticojejunostomy.

## Conclusions

We report a case of Petersen’s hernia observed in a LT patient. Petersen’s hernia is a relatively rare complication after LT. However, it should be considered as a cause of bowel obstruction in recipients with Roux-en-Y reconstruction. Early diagnosis and prompt and proper surgical intervention including second-look surgery are crucial for the treatment of this type of hernia.

## Abbreviations

CT: Computed tomography
LDLT: Living donor liver transplantation
LT: Liver transplantation
PTBD: Percutaneous transhepatic biliary drainage
